# Cold-induced vasodilation during continuous exercise in the extreme cold air (-30.6 °c)

**DOI:** 10.1186/2046-7648-4-S1-A58

**Published:** 2015-09-14

**Authors:** Chuansi Gao, Li-Yen Lin, Amitava Halder, Kalev Kuklane

**Affiliations:** 1 Thermal Environment Laboratory, Division of Ergonomics and Aerosol Technology, Department of Design Sciences, Faculty of Engineering, Lund University, Box 118, 221 00 Lund, Sweden; 2 Department of Testing and Certification, Taiwan Textile Research Institute (TTRI), 6 Chengtian Road, Tucheng District, New Taipei, 23674, Taiwan

## Introduction

Cold induced vasodilation (CIVD) in previous studies was mostly evoked by cold water immersion at 0, 5, and 8 °C of the upper or lower extremities without performing physical work [1]. A limited number of investigations incorporated intermittent exercises during cold air exposure [2]. Literature has documented that the CIVD occurrence depended on the body core temperature [2], [3]. It was observed that CIVD in cold water immersion was highly variable across the fingers and was not a generalizable response across fingers or toes [4]. However, the number of studies on cold air induced vasodilation in the extremely cold is limited. The objective of this study was to investigate individual variations of finger CIVD in relation to the core and mean skin temperatures during continuous exercise in the extreme cold air (-30.6 °C).

## Methods

Four young and healthy male subjects wore cold protective clothing ensemble (*I_cl _*= 1.89 clo) and walked at 4 MET (232.8 W/m^2^) on a treadmill in a climatic chamber (*T_a_*= -30.6 C, *V_a_*= 0.4 m/s) for 90 min [5]. Hestra wind stopper fleece fabric gloves (relatively thin) were used. The core and skin temperatures were measured respectively in the rectum 10 cm above the anal sphincter, and on the forehead and left side of the body on the upper arm, forearm, hand, little finger, chest, scapula, thigh, calf, and little toe. The mean skin temperature (*T_sk_*) was calculated below.

Tsk=0.07(T_forehead+T_upperarm+T_forearm)+0.175(T_chest+T_scapula)+0.05T_hand+0.19T_thigh+0.20T_calf

## Results

CIVD in the little finger occurred when the subjects' rectal temperatures (T_re_) were relatively stable in the range of 37.1 - 38.1 °C and the T_sk _in the range of 32.0 - 25.3 °C. Within these ranges, the finger CIVD periodical responses were not dependent on the T_re _and T_sk _changes. The onset time, T_min_, T_max _and T_finger_mean_, amplitude (T_max _- T_min_), frequency (number of waves) of the CIVD were 14.6 (3.5) min, 3.8 (3.4) °C, 16.5 (3.6) °C, 7.9 (1.4) °C, 12.7 (4.4) °C, 7.5 (4.7) respectively.

## Discussion

The finger CIVD appeared in all four subjects. However, the onset time, minimum, maximum and mean finger temperatures, amplitude, frequency of the CIVD varied among the four subjects. The finger CIVD occurred when the mean finger temperatures of the four subjects were below 10 °C, thus it seems to be related to the local cooling of the extremities during continuous and stable exercise at high metabolic rate in the extremely cold environment. The CIVD in toes was not as clear as in the fingers, which might be attributed to the continuous walking.

## Conclusion

The finger CIVD varies among the subjects. Its occurrence is not dependent on T_re _and T_sk _changes within the T_re _and T_sk _ranges (37.1 - 38.1 °C and 32.0 - 25.3 °C), but it is associated with the local cooling of the extremities during continuous 90 min walking at 4 MET in the extreme cold air (-30.6 °C).
